# Respiratory resistance and reactance in adults with sickle cell anemia: Part 2—Fractional-order modeling and a clinical decision support system for the diagnosis of respiratory disorders

**DOI:** 10.1371/journal.pone.0213257

**Published:** 2019-03-07

**Authors:** Cirlene de Lima Marinho, Maria Christina Paixão Maioli, Jorge Luis Machado do Amaral, Agnaldo José Lopes, Pedro Lopes de Melo

**Affiliations:** 1 Biomedical Instrumentation Laboratory—Institute of Biology and Faculty of Engineering, and BioVasc Research Laboratory—Institute of Biology, State University of Rio de Janeiro, Rio de Janeiro, Brazil; 2 Department of Hematology—Faculty of Medical Sciences, State University of Rio de Janeiro, Rio de Janeiro, Brazil; 3 Department of Electronics and Telecommunications Engineering, State University of Rio de Janeiro, Rio de Janeiro, Brazil; 4 School of Medical Sciences, Pulmonary Function Testing Laboratory, Rio de Janeiro/RJ, State University of Rio de Janeiro, Rio de Janeiro, Brazil; 5 Rehabilitation Sciences Post-Graduation Program, Augusto Motta University Centre, Rio de Janeiro, Brazil; University of Alabama at Birmingham, UNITED STATES

## Abstract

**Background:**

A better understanding of sickle cell anemia (SCA) and improvements in drug therapy and health policy have contributed to the emergence of a large population of adults living with this disease. The mechanisms by which SCA produces adverse effects on the respiratory system of these patients are largely unknown. Fractional-order (FrOr) models have a high potential to improve pulmonary clinical science and could be useful for diagnostic purposes, offering accurate models with an improved ability to mimic nature. Part 2 of this two-part study examines the changes in respiratory mechanics in patients with SCA using the new perspective of the FrOr models. These results are compared with those obtained in traditional forced oscillation (FOT) parameters, investigated in Part 1 of the present study, complementing this first analysis.

**Methodology/Principal findings:**

The data consisted of three categories of subjects: controls (n = 23), patients with a normal spirometric exam (n = 21) and those presenting restriction (n = 24). The diagnostic accuracy was evaluated by investigating the area under the receiver operating characteristic curve (AUC). Initially, it was observed that biomechanical changes in SCA included increased values of fractional inertance, as well as damping and hysteresivity (p<0.001). The correlation analysis showed that FrOr parameters are associated with functional exercise capacity (R = -0.57), pulmonary diffusion (R = -0.71), respiratory muscle performance (R = 0.50), pulmonary flows (R = -0.62) and airway obstruction (R = 0.60). Fractional-order modeling showed high diagnostic accuracy in the detection of early respiratory abnormalities (AUC = 0.93), outperforming spirometry (p<0.03) and standard FOT analysis (p<0.01) used in Part 1 of this study. A combination of machine learning methods with fractional-order modeling further improved diagnostic accuracy (AUC = 0.97).

**Conclusions:**

FrOr modeling improved our knowledge about the biomechanical abnormalities in adults with SCA. Changes in FrOr parameters are associated with functional exercise capacity decline, abnormal pulmonary mechanics and diffusion. FrOr modeling outperformed spirometric and traditional forced oscillation analyses, showing a high diagnostic accuracy in the diagnosis of early respiratory abnormalities that was further improved by an automatic clinical decision support system. This finding suggested the potential utility of this combination to help identify early respiratory changes in patients with SCA.

## Introduction

The term sickle cell disease (SCD) is associated with a group of inherited red blood cell disorders. People with SCD have abnormal hemoglobin, called hemoglobin S or sickle-shaped hemoglobin, in their red blood cells. The most severe type of SCD is sickle cell anemia (SCA). This disease originated due to a mutation that protected the population from a Malaria epidemic [1]. The erythrocytes of these individuals undergo structural changes, adopting a sickle shape that confers high morbidity and mortality [2]. This disease is one of the most prevalent disorders among existing hereditary diseases, affecting approximately 300,000 children annually [3–5]. These irregularly shaped cells can get stuck in small blood vessels, introducing abnormal repercussions in various organs due to the reduction or even blockage of blood flow. Pulmonary complications account for the largest proportion of deaths among adults with SCA [6]. The lungs of these patients are frequently affected by acute thoracic syndrome (ATS), which correlates with pulmonary wheezing and cognitive dysfunction due to vaso-occlusion of the capillaries that supplement the brain tissue [7–12]. Thus, the early diagnosis of ATS is fundamental for reversing unfavorable clinical outcomes [7].

Due to the dramatic improvement in SCD care over the last decades, associated with newborn screening, penicillin prophylaxis, primary stroke prevention, and hydroxyurea treatment, life expectancy has improved significantly. The childhood mortality is now close to that in the general population, with an observed median survival of more than 60 years in high income countries [5]. Thus, the number of patients with SCD is expected to increase [6], and the emergence of such a large population of adults living with SCA demands further understanding of the overall changes in their respiratory function.

Spirometric and plethysmographic tests are usually used to evaluate patients with SCA. However, these exams demand an understanding of reliable forced expiratory maneuvers [13]. The performance of these tests in SCA may be difficult due to the usual presence of cognitive deficiency in these patients. This limitation may result in the under diagnosis of pulmonary abnormalities in a timely manner, compromising adequate follow-up and treatment [14].

The forced oscillation technique (FOT) allows us to measure respiratory mechanics, including resistance and reactance, noninvasively during normal tidal breathing. Instead of using the respiratory muscles as the source of force, this method superimposes oscillations onto spontaneous breathing using an external loudspeaker. Particularly in SCA, which has been linked to impaired cognitive function [15], the fact that this technique does not require complex panting maneuvers is a significant advantage. Another important characteristic is that FOT provides information about lung mechanics that cannot be obtained using the classical pulmonary function tests. In this sense, fractional-order (FrOr) modeling is increasingly used to interpret FOT measurements. These models have a high potential to improve pulmonary clinical science [16, 17] and could be useful for diagnostic purposes, offering parsimonious yet accurate models with an improved ability to mimic nature [18]. Recently, new FrOr models were introduced [19–23] and are especially useful for the clinical analysis of several respiratory diseases [17], including children with asthma [22] and cystic fibrosis [23], as well as in patients with chronic obstructive pulmonary disease (COPD) [20, 21]. Further studies from our group have provided additional evidence that FrOr models may contribute to the early identification of mild lung abnormalities in adults with asthma [24] and asbestos-exposed workers [25] and the detection of the early effects of COPD [26]. Despite several attractive characteristics of the FrOr models, they have not been widely used in clinical practice. One of the major limitations is the difficulty encountered by pulmonologists in interpreting the resulting FrOr parameters because the physiological or clinical meaning of the derived parameters is not clear.

The six-minute walk test (6MWT) is widely used to assess functional exercise capacity. The 6MWT evaluates the integrated response of all of the organs and systems involved in exercise, including the lungs, heart, circulatory and neuromuscular systems [27], closely reflecting the activities of daily living. Therefore, the 6MWT has the potential to increase our knowledge concerning the relationship between FrOr parameters and functional exercise capacity, helping to elucidate the physiological or clinical meaning of these parameters. In this context, important questions have recently arisen [18]: what does the fractional-order dynamic behavior tell us, and what is the link in the underlying structure and function of the systems that produce them? Although the 6MWT may help to answer this question, to the best of our knowledge, there are no reports in the literature focusing on these associations.

Part 1 of this two-part study (presented in the December 2017 issue of PLoS ONE) contributed to improve our knowledge about the respiratory abnormalities in SCA using the FOT [28]. This study also evaluated the associations of FOT with the functional exercise capacity and investigated the early detection of respiratory abnormalities using traditional FOT parameters associated with machine learning (ML) methods. This association achieved adequate diagnostic accuracy, suggesting the potential utility of these methods as markers of early respiratory abnormalities in patients with SCA. However, this association was not enough to diagnose respiratory abnormalities in SCA with a high accuracy.

Part 2 of this study provides a FrOr analysis from the same dataset of SCA patients, which complements and deepens the analysis described in Part 1 [28]. In this context, we initially examine the changes in respiratory mechanics in patients with SCA using the new perspective of the FrOr model. Then, we investigate the association between this model and changes in diffusing capacity, respiratory muscle performance and functional exercise capacity. Finally, we evaluate the diagnostic accuracy of FrOr parameters in the early diagnosis of respiratory abnormalities in patients with SCA. These results are compared with those obtained in traditional FOT parameters, investigated in Part 1 of the present study [28].

## Methods

### Subjects, pulmonary function, 6MWT measurements and machine learning algorithm

The Research Ethics Committee of the Pedro Ernesto University Hospital (HUPE) approved the study, which was registered at ClinicalTrials.gov (identifier: NCT02565849) and obeys the Declaration of Helsinki. A detailed description of the methods, including the flowchart of the study, pulmonary function, reference ranges, FOT and 6MWT measurements and the machine learning algorithms evaluated are presented in Part 1 of the present study [28] and will not be repeated here for the sake of simplicity.

### Statistical analysis

Briefly, the results are present as the mean±SD. Initially, the sample distribution characteristics were assessed using Shapiro-Wilk’s test. A one-way ANOVA with Tukey’s test was performed to analyze the normally distributed data; conversely, a non-parametric analysis (Kruskal-Wallis) with a Mann-Whitney test was performed for the non-normally distributed data. Differences with p≤0.05 were considered statistically significant. These analyses were performed using Origin 8.0 (Microcal Software Inc., Northampton, Massachusetts, United States).

Correlations were studied using Pearson`s correlation coefficient in the presence of normal distributions, while Spearman’s correlation was used in non-normal distributions. These correlations were classified as follows [29]:

- Small or no correlation: between 0 and 0.25 (or -0.25);
- Reasonable correlation: between 0.25 and 0.50 (or -0.25 to -0.50);
- Moderate to good correlation: between 0.50 and 0.75 (or -0.50 to -0.75);
- Very good to excellent correlation: greater than 0.75 (or -0.75).

As several correlations were computed, we performed a correction in the significance level to minimize the chances of making a Type I error. We used a modified Bonferroni approach, which requires dividing usual p-value by an estimate of the effective number of independent correlations used [30]. FOT describes resistive and reactive properties, thus, two independent variables were considered. In general, four independent variables are observed in other exams, which results in eight independent correlations and a corrected significance level for correlation analysis of 0.0063 (0.05/8).

The clinical potential of the FOT indexes in the detection of respiratory alterations was investigated using receiver operation characteristic (ROC) analysis. The values of sensitivity, specificity, and area under the curve (AUC) were obtained based on the optimal cut-off point, as determined by the ROC curve analysis. According to the literature, ROC curves with AUCs between 0.50 and 0.70 indicate low diagnostic accuracy, AUCs between 0.70 and 0.90 indicate moderate accuracy, and AUCs between 0.90 and 1.00 indicate high accuracy [31, 32]. Goedhart et al. [33] considered 0.7 to be a good cut-off value for a useful discriminator for clinical use. In the present study, we considered 0.75 to be the minimum value of the AUC for adequate diagnostic accuracy. The ROC analyses were conducted using MedCalc 12 (MedCalc Software, Mariakerke, Belgium). This part of the study follows the STARD requirements for studies of diagnostic accuracy [34].

The sample size was calculated based on the criteria of the comparison of the area under a ROC curve with a null hypothesis value. The aim was to show that an AUC of 0.75, describing adequate diagnostic accuracy [33], was significantly different from the null hypothesis value of 0.5, which indicates no discriminating power. This analysis was performed based on the results obtained in a pilot study including 14 controls and 14 patients using MedCalc 13 (MedCalc Software, Mariakerke, Belgium), according to the theory described by Hanley and McNeil [35]. A type I error of 0.10 and a type II error of 0.10 were assumed as adequate, which resulted in a minimum of 20 volunteers per group.

### Fractional-order modeling

The model used in the present work was proposed recently by Ionescu et al. [22], and it was the most sensitive model observed in a previous study in asthma [24]. The parameters of the respiratory impedance (Z_FrOr_) fractional model were estimated according to previous works [24, 25], including a frequency-dependent fractional-order inertance (FrL) and a fractional inertance coefficient (α):

$$Z_{FrOr}(j\omega )={FrL(j\omega )}^{\alpha }+\frac{1}{FrC{\left(j\omega \right)}^{\beta }}$$ (1)

The fractional-order values and coefficients will change according to the properties (morphology, geometry) of the respiratory system [17]. FrL describes the joint effect of the resistive and inertial properties of the airways. The degree of the influence of FrL in the frequency dependence of airway resistance and inertance is related with the α coefficient. Lower values of α are related with an increased influence of FrL in the airway resistance and a reduced influence in airway inertance. In respiratory impedance curves it reflects increased resistance and more negative values of reactance in higher frequencies. The model also includes a more peripheral component described as the constant-phase fractional-order compliance (*FrC)* associated with a fractional compliance coefficient (0 ≤ *β* ≤ 1). Lower values of β are related with an increased influence of FrC in the resistance and a reduced influence in compliance. In respiratory impedance curves it reflects increased resistance and more negative values of reactance in lower frequencies.

These results were interpreted physiologically using the damping (*G*), elastance (*H*) and the hysteresivity coefficient (*η*) as described by the following equations:

$$G=\frac{1}{C}cos\left(\frac{\pi }{2}\beta \right)$$ (2)

$$H=\frac{1}{C}sin\left(\frac{\pi }{2}\beta \right)$$ (3)

$$\eta =\frac{G}{H}$$ (4)

Damping is a measure of the energy dissipation in the respiratory tissues [17], while elastance is a measure of potential elastic energy accumulation. Hysteresivity is a concept that addresses the heterogeneity of ventilation in the lung, with greater values often associated with more heterogeneity [17].

Curve fitting of the FrOr model was performed using the ModeLIB program, which was also developed in our laboratory. This program employs the Levenberg-Marquardt algorithm to determine the set of parameters of the model that best represents the input dataset in terms of least squares. Together with the model estimates, this analysis also provides the calculated total error value (MSEt), an overall measure of the “goodness of fit” of the model. The square root of the sum of the real (MSEr) and imaginary (MSEx) impedance estimation errors is used for this purpose.

## Results

The clinical, biometrical and spirometric characteristics of the studied subjects were described in Part 1 of the present study [28], which also described plethysmographic and pulmonary diffusion results. Inspiratory pressures, expiratory pressures and functional exercise capacities were also described previously.

**Fig 1:**
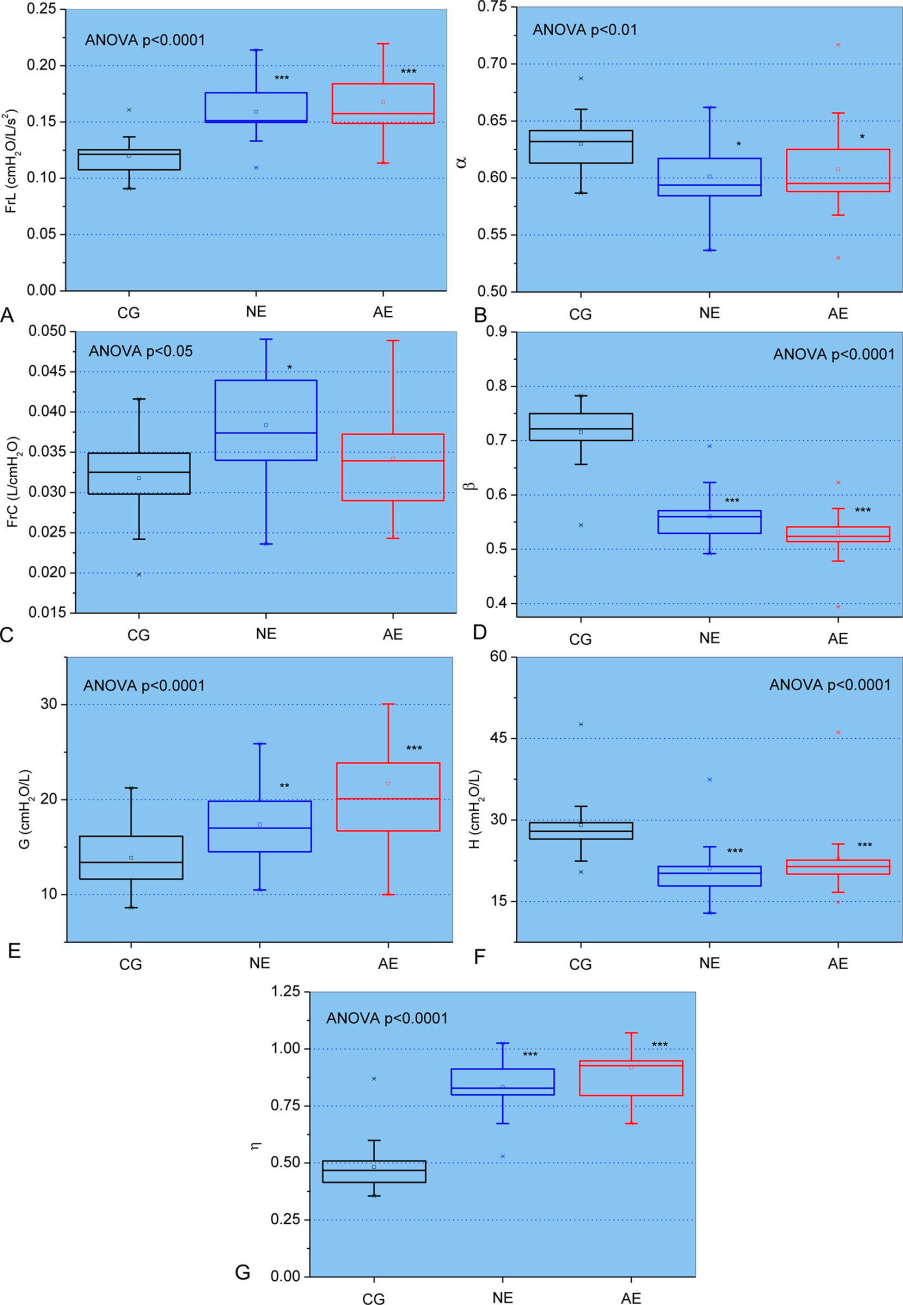
Changes of the parameters obtained from the fractional-order model in the control group and patients with normal (NE) and abnormal (AE) spirometry: Fractional inertance (FrL; A) and associated fractional-order angle (α; B), the fractional compliance (FrC; C) and associated fractional-order angle (β; D), respiratory damping (G; E), elastance (H; F) and hysteresivity (η; G). The top and the bottom of the box plot represent the 25th- to 75th-percentile values while the circle represents the mean value, and the bar across the box represents the 50th-percentile value. The whiskers outside the box represent the 10th-to 90th-percentile values. *p<0.05, **p<0.01 and ***p<0.001 related to the control group.

Fig 1 describes the results of the FrOr modeling. The progression of respiratory involvement in SCA resulted in highly significant increases in FrL, G and η (p<0.0001, Fig 1A, 1E and 1G, respectively) while FrC showed a significant increase (p<0.05, Fig 1C). Additionally, α presented a significant decrease with airway obstruction in SCA (p<0.01; Fig 1B). β and H also showed a significant reduction (p<0.0001; Fig 1D and 1F, respectively).

Table 1 shows that FrL presented the best correlations among spirometric and FrOr parameters [R = -0.62, forced expiratory flow between 25% and 75% of the forced vital capacity—FEFmax (%)] and that FrOr parameters were not correlated with the ratio of the forced expiratory volume in the first second and the forced vital capacity (FEV_1_/FVC). The degrees of association were reasonable to good.

**Table 1:** Correlation analysis among fractional-order parameters and spirometric results. Significance was analyzed after Bonferroni correction. The highest associations are described in bold.

	FEV_1_ (L)	FEV_1_ (%)	FVC (L)	FVC (%)	FEV_1_/FVC	FEF max (L)	FEF max (%)
FrL	-0.51 <0.0001	-0.49 <0.0001	-0.49 <0.0001	-0.54 <0.0001	-0.08 ns	-0.58 <0.0001	**-0.62** <0.0001
α	-0.22 ns	0.001 ns	-0.27 ns	-0.04 ns	0.09 ns	-0.26 Ns	0.002 ns
FrC	0.44 <0.0005	0.11 ns	**0.45** 0.0001	0.13 ns	0.01 ns	0.09 Ns	-0.20 ns
β	0.42 <0.0005	0.53 <0.0001	0.38 0.001	**0.56** <0.0001	0.15 ns	0.31 Ns	0.49 <0.0001
G	**-0.60** <0.0001	-0.49 <0.0001	-0.57 <0.0001	-0.52 <0.0001	-0.19 ns	-0.30 Ns	-0.23 ns
H	-0.27 ns	0.02 ns	-0.28 ns	0.02 ns	-0.01 ns	-0.02 Ns	0.30 ns
η	-0.45 0.0001	**-0.54** <0.0001	-0.40 <0.001	-0.57 <0.0001	-0.17 ns	-0.31 Ns	-0.47 <0.0001

FEV1: forced expiratory volume in the first second; FVC: forced vital capacity; FEF: forced expiratory flow between 25% and 75% of the FVC; %: percentage of the predicted values. FrL: fractional-order inertance; α: fractional inertance coefficient; FrC: fractional-order compliance; β: fractional compliance coefficient; G: damping factor; H: elastance; η: hysteresivity coefficient.

The relationships among plethysmographic and FrOr parameters are described in Table 2. Reasonable to good associations were observed, and FrL presented the best correlations, showing a good direct association with airway resistance (Raw; R = 0.60).

**Table 2:** Correlation analysis among fractional-order parameters and volumetric results. Significance was analyzed after Bonferroni correction. The highest associations are described in bold.

	TLC (L)	TLC (%)	FRC (L)	FRC (%)	RV (L)	RV (%)	RV/TLC	RV/TLC (%)	Raw
FrL	-0.34 <0.005	-0.51 <0.0001	-0.35 <0.005	-0.32 ns	0.02 ns	-0.12 ns	0.44 <0.0005	0.42 <0.0005	**0.60** <0.0001
α	-0.12 ns	0.06 ns	-0.12 ns	0.05 ns	0.08 ns	-0.11 ns	0.29 ns	0.13 ns	0.03 ns
FrC	0.29 ns	0.04 ns	**0.34** <0.005	-0.02 ns	0.03 ns	0.18 ns	-0.31 ns	-0.13 ns	0.03 ns
β	0.27 ns	**0.59** <0.0001	0.35 <0.005	0.35 <0.005	0.05 ns	0.15 ns	-0.27 ns	-0.35 <0.005	-0.49 <0.0001
G	-0.28 ns	**-0.46** <0.0001	-0.43 <0.0005	-0.17 ns	0.02 ns	-0.35 <0.005	0.45 0.0001	0.34 <0.005	0.33 ns
H	-0.14 ns	0.10 ns	-0.17 ns	0.15 ns	0.03 ns	-0.17 ns	0.23 ns	0.02 ns	-0.15 ns
η	-0.26 ns	**-0.59** <0.0001	-0.36 <0.005	-0.34 <0.005	-0.04 ns	-0.19 ns	0.29 ns	0.35 <0.005	0.47 <0.0001

TLC: total lung capacity; FRC: functional residual capacity; RV: residual volume; Raw airway resistance.

The associations between FrOr and pulmonary diffusion parameters presented reasonable to good values (Table 3). FrOr parameters were not correlated with the ratio of carbon monoxide diffusion capacity and alveolar volume (DLCO/AV). Table 3 also shows that FrL, β and η presented the best correlations among FrOr parameters [R = 0.71, DLCO percentage of the predicted values without correction (DLCOa%)] while G showed a good inverse correlation with AV (%).

**Table 3:** Correlation analysis among fractional-order parameters and pulmonary diffusion capacity results. Significance was analyzed after Bonferroni correction.The highest associations are described in bold.

	DLCOa	DLCOa (%)	DLCOc	DLCOc (%)	DLCO/AVc	DLCO/AVc (%)	AV (L)	AV (%)
FrL	-0.65 <0.0001	**-0.71** <0.0001	-0.43 <0.0005	-0.51 <0.0001	-0.06 ns	-0.11 ns	-0.51 <0.0001	-0.63 <0.0001
α	-0.17 ns	0.07 ns	-0.29 ns	-0.01 ns	-0.17 ns	-0.06 ns	-0.26 ns	-0.05 ns
FrC	0.22 ns	-0.07 ns	0.31 ns	0.00 ns	-0.04 ns	-0.08 ns	**0.41** 0.0005	0.14 ns
β	0.51 <0.0001	**0.71** <0.0001	0.23 ns	0.51 <0.0001	-0.16 ns	-0.03 ns	0.39 <0.001	0.65 <0.0001
G	-0.54 <0.0001	-0.51 <0.0001	-0.42 <0.0005	-0.44 <0.0005	0.01 ns	-0.02 ns	-0.54 <0.0001	**-0.57** <0.0001
H	-0.08 ns	0.23 ns	-0.24 ns	0.10 ns	-0.10 ns	0.00 ns	-0.24 ns	0.06 ns
η	-0.51 <0.0001	**-0.71** <0.0001	-0.25 ns	-0.52 <0.0001	0.15 ns	0.02 ns	-0.41 <0.001	-0.65 <0.0001

DLCO: carbon monoxide diffusion capacity; AV: alveolar volume; diffusion coefficient (DLCO/AV); a: values without correction; c: corrected for the concentration level of hemoglobin; %: percentage of the predicted values.

FrL, G and η presented reasonably significant inverse associations with maximum expiratory pressure (Pe) while β was directly associated with Pe (Table 4). In contrast, α, FrC, and H did not present significant correlations with respiratory pressures.

**Table 4:** Correlation analysis among fractional-order parameters and respiratory muscle pressure. Significance was analyzed after Bonferroni correction. The highest associations are described in bold.

	Pi	Pi (%)	Pe	Pe (%)
FrL	0.23 ns	-0.27 ns	**-0.48** <0.0001	-0.44 <0.0005
α	0.02 ns	-0.06 ns	-0.10 ns	0.10 ns
FrC	-0.10 ns	0.10 ns	0.17 ns	-0.07 ns
β	-0.25 ns	0.23 ns	0.41 0.0005	**0.50** <0.0001
G	0.18 ns	-0.20 ns	**-0.34** <0.005	-0.25 ns
H	-0.01 ns	-0.03 ns	-0.02 ns	0.20 ns
η	0.24 ns	-0.22 ns	-0.40 <0.001	**-0.46** <0.0001

Pi: maximal inspiratory pressure; Pe: maximum expiratory pressure.

Table 5 describes the associations between the FrOr and functional exercise capacity. FrL was associated with the Final Borg Scale, while β was correlated with final peripheral oxygen saturation (SpO_2_) and final Borg scale. Additionally, G presented a significant degree of association with 6MWT and final SpO_2_. Among the FrOr parameters, the highest degree of association with the functional exercise capacity parameters was between η and final SpO_2_ (R = -0.57). In the first part of this study [28], the maximum values of correlation were 0.55, 0.48, and -0.38, for spirometry, plethysmography and traditional FOT parameters, respectively.

**Table 5:** Correlation analysis among fractional-order parameters and 6MWT results. Significance was analyzed after Bonferroni correction. The highest associations are described in bold.

	6MWT	6MWT (%)	RR Initial	RR Final	SpO_2_ Initial	SpO_2_ Final	Borg Scale Initial	Borg Scale Final
FrL	-0.30 ns	-0.23 ns	0.23 ns	0.30 ns	-0.23 ns	-0.29 ns	0.04 ns	**0.36** <0.005
α	-0.06 ns	0.09 ns	-0.12 ns	0.01 ns	0.29 ns	0.25 ns	-0.18 ns	-0.06 ns
FrC	0.11 ns	-0.11 ns	0.06 ns	-0.03 ns	-0.08 ns	-0.03 ns	-0.07 ns	0.07 ns
β	0.27 ns	0.29 ns	-0.35 <0.005	-0.31 ns	0.50 <0.0001	**0.56** <0.0001	-0.17 ns	-0.41 <0.001
G	**-0.38** <0.005	-0.25 ns	0.19 ns	0.22 ns	-0.30 ns	**-0.38** <0.005	0.03 ns	0.28 ns
H	-0.11 ns	0.09 ns	-0.15 ns	-0.06 ns	0.25 ns	0.21 ns	-0.14 ns	-0.15 ns
η	-0.29 ns	-0.30 ns	0.35 <0.005	0.30 ns	-0.51 <0.0001	**-0.57** <0.0001	0.16 ns	0.41 0.0005

6MWT: Six-minute walk test distance; (%): predicted percentage; RR: Respiratory rate; SpO2: Peripheral oxygen saturation.

Four of the studied FrOr parameters showed high sensitivity to detect changes in the presence of normal spirometric exams (FrL, β, H, η; Table 6). These parameters also showed adequate diagnostic performance in the initial ROC analysis of patients with abnormal spirometric exams.

**Table 6:** Diagnostic accuracy of the fractional-order parameters in the detection of respiratory alterations in patients with sickle cell disease. Values obtained in patients with normal values in the spirometric exam and abnormal spirometry.

	FrL	α	C	β	G	H	η
Normal exam							
AUC	**0.910**	0.786	0.763	**0.967**	0.743	**0.892**	**0.967**
Se (%)	90.48	66.67	76.19	95.24	76.19	90.48	95.24
Sp (%)	91.30	82.61	65.22	95.65	69.57	91.30	95.65
Cut-off	>0.137	≤0.606	>0.033	≤0.623	>14.098	≤25.06	>0.599
Abnormal exam							
AUC	**0.918**	0.731	0.624	**0.989**	0.859	**0.850**	**0.989**
Se (%)	87.50	58.33	66.67	100.00	70.83	79.17	100.00
Sp (%)	91.30	86.96	65.22	95.65	95.65	91.30	95.65
Cut-off	>0.137	≤0.598	>0.033	≤0.623	>18.341	≤24.931	>0.599

AUC: area under the receiver-operator curve; Se: sensibility; Sp: specificity.

**Fig 2:**
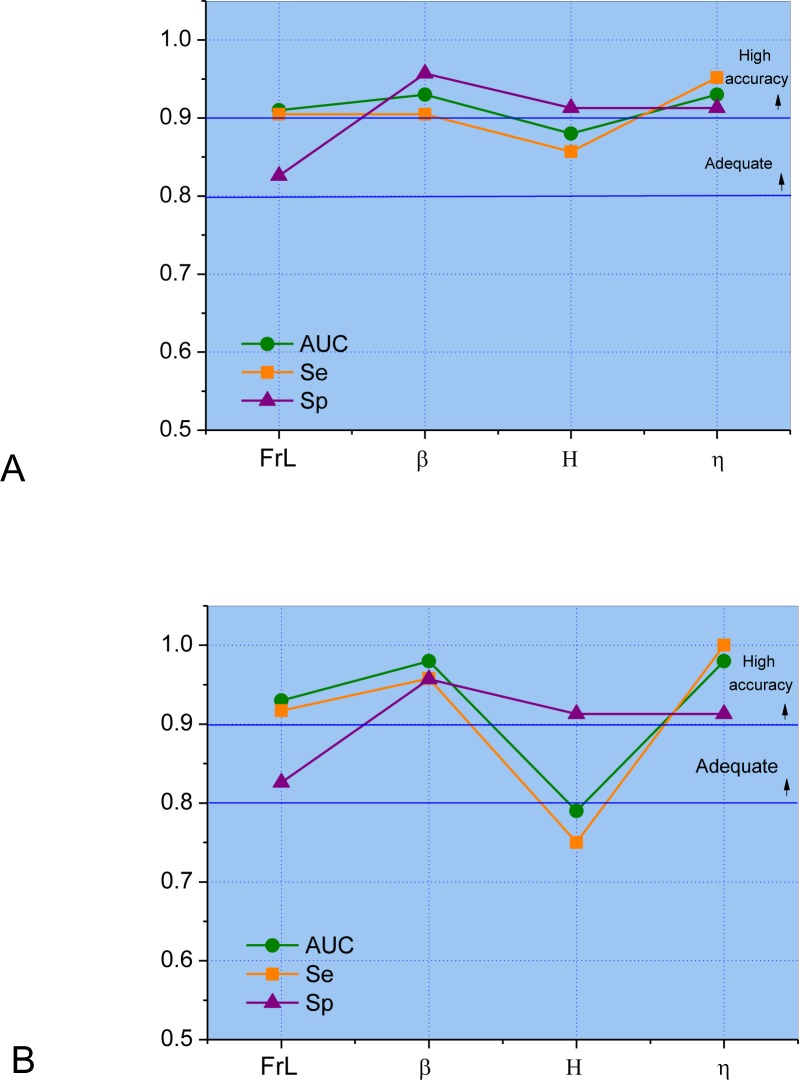
Leave-one-out cross-validation analysis performed in the most discriminative parameters described in Table 6 in the presence of normal spirometric exams (A) and abnormal spirometry (B).

Leave-one-out cross-validation (LOOCV) analysis [36] performed in the most discriminative parameters described in Table 6 showed a high diagnostic accuracy for FrL, β, and η in the presence of normal spirometric exams (Fig 2A). These parameters also presented a high diagnostic accuracy (AUC>0.9) in patients with abnormal spirometry (Fig 2B).

**Fig 3:**
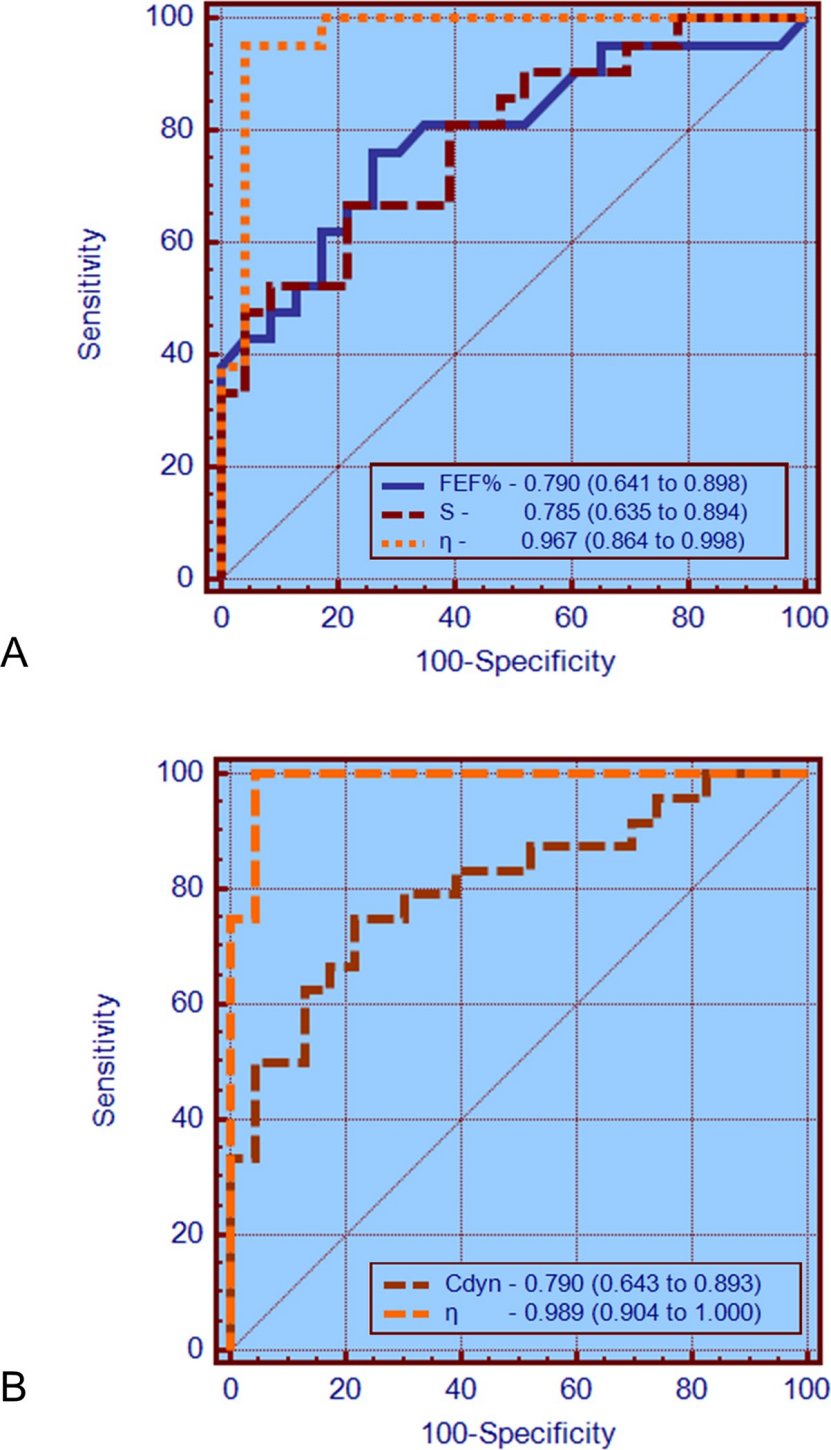
ROC curves, AUCs and the 95% confidence interval for the most accurate parameters observed in spirometry, classical FOT analysis and for the FrOr model in patients with normal exams (A) and with abnormal spirometric exams (B). The AUCs of FEF% and S were similar in the NE group (p = ns) and η showed a significantly higher AUC than FEF% (p<0.03) and S (p = 0.01). In patients with abnormal spirometric exams (B), η had a significantly higher AUC than the best traditional FOT parameter (Cdyn, p = 0.005).

Comparing the ability of the best parameters from spirometry, traditional FOT and FrOr to identify initial respiratory changes in SCD (Fig 3), the AUCs of FEF% and the slope of the resistance values (S) were similar in the NE group (p = ns). In contrast, η showed a significantly higher AUC than FEF% (p<0.03) and S (p = 0.01). In patients with abnormal spirometric exams (Fig 3B), η had a significantly higher AUC than the best traditional FOT parameter [dynamic compliance (Cdyn), p = 0.005].

**Fig 4:**
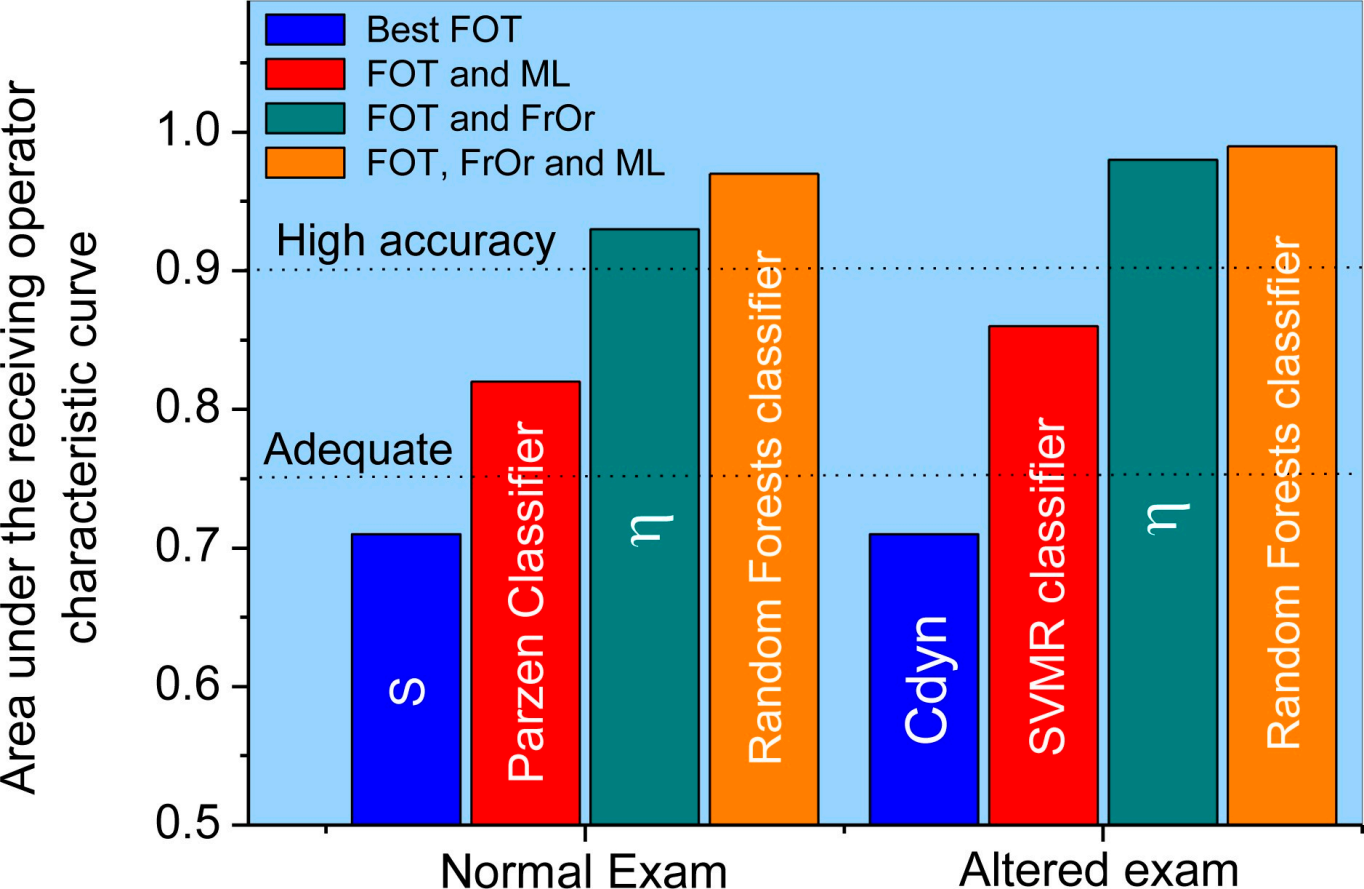
Results considering the more restrictive analysis using leave-one-out cross-validation in the best FOT parameter and FOT associated with machine learning methods (obtained in the first part of this research [28]). These results are compared with those obtained in the present study using FrOr modeling and FrOr modeling combined with machine learning methods in patients with normal (A) and abnormal (B) spirometry.

However, a more restrictive analysis using leave-one-out cross-validation in the first part of this research [28] showed that none of these parameters reached adequate values for clinical use. The use of machine learning methods resulted in an improvement in the diagnostic accuracy (Fig 4). Interestingly, in the present study, the improvement in diagnostic accuracy using FrOr modeling was higher than that obtained using ML methods, allowing us to achieve high accuracy (Fig 4).

The analysis of the diagnostic accuracy combining ML methods and all FrOr parameters is described in Table 7. Table 8 shows the results obtained using an exhaustive search of the best FrOr parameters. The use of cross products did not improve the results, even when a search for the best parameters in the cross products was performed.

**Table 7:** Evaluation of the diagnostic accuracy of the machine learning algorithms using all FrOr parameters in detecting respiratory alterations in patients with sickle cell anemia and normal and abnormal spirometric exams.

	SVML [37, 38]	ADAB [39]	1-NN [37, 38, 40]	RF [39, 40]	SVMR [40]	PARZEN [41]
Normal exam						
AUC	0.92	0.82	**0.96**	0.95	0.92	0.90
Se (%)	95.2	85.7	95.2	95.2	95.2	81.0
Sp (%)	95.7	82.6	95.7	95.7	95.7	95.7
Abnormal exam						
AUC	0.96	0.92	**0.97**	0.96	0.90	0.89
Se (%)	100.0	100.0	100.0	100.0	97.5	91.7
Sp (%)	95.7	82.6	95.7	95.7	91.3	95.7

SVML: Support Vector Machine with Linear Kernel

ADAB: Adaboost with decision tree classifiers

1-NN: K Nearest Neighbor (K = 1)

RF: Random Forests

SVMR: Support Vector Machine with Radial Basis Kernel

PARZEN: Parzen classifier

**Table 8:** Evaluation of the diagnostic accuracy of the machine learning algorithms using an exhaustive search of the best FrOr parameters in detecting respiratory alterations in patients with sickle cell anemia and normal spirometric exams.

	SVML	ADAB	1-NN	RF	SVMR	PARZEN
Normal exam						
AUC	0.95	0.89	0.95	**0.97**	0.95	0.95
Se (%)	95.2	95.2	95.2	95.2	95.2	95.2
Sp (%)	95.7	91.3	95.7	95.7	95.7	91.3
Parameters	(FrL,β)	(α,η)	(β,G,η)	(FrL,G,η)	(L,β)	(L,β,η)
Abnormal exam						
AUC	0.97	0.95	0.96	**0.99**	0.93	0.98
Se (%)	100.0	91.7	100.0	100.0	87.5	91.7
Sp (%)	95.7	87.0	95.7	95.7	95.7	100.0
Parameters	(FrL,β)	(FrL,G,η)	(β,G,η)	(β,G,η)	(FrL,α,C,G,η)	(β)

## Discussion

To the best of our knowledge, this is the first study to investigate the concept of fractional order modeling of respiratory impedance in adults with SCA. The major findings are that (1) adults with SCA showed increased fractional inertance, damping and hysteresivity when compared with community controls, 2) fractional-order parameters are correlated with functional exercise capacity decline and pulmonary diffusion abnormalities, 3) fractional-order analysis outperformed standard FOT, as well as FOT measurements associated with machine learning methods, in the detection of early abnormalities, and 4) fractional-order analysis combined with machine learning methods further improved diagnostic accuracy, allowing us to attain a high accuracy in the detection of early respiratory abnormalities in patients with SCA.

Recently, the concept of FrOr modeling of the respiratory system has received significant interest in the research community [18, 22, 24, 42, 43]. Theoretically, these emerging models have an improved sensitivity to pathologic changes, due to an improved ability to capture the characteristics of the respiratory mechanics.

FrL in controls presented small values (Fig 1A), similar to previous studies [26]. This parameter increased in SCA patients, which is consistent with the interpretation that resistive properties are captured, at least in part, by the real component of the FrL term [21]. Additional support to this hypothesis is provided by the findings of inverse good correlations with spirometric indexes of airway obstruction (Table 1), and the highest association with airway resistance measured with the plethysmograph (Table 2). Another interesting finding was the presence of inverse good correlations with diffusion capacity indexes (Table 3) and reasonable associations with respiratory pressures (Table 4). In close agreement with the involved physiology, the increase in FrL was associated with an increase in Final Borg Scale (Table 5).

The values of α were slightly reduced in patients with SCA (Fig 1B). This finding is consistent with the observation that in the SCA subjects there is a negative dependence of the resistance as a function of frequency (Fig 3A in the first part of this study [28]). It is also consistent with a recent work in asbestosis [25], a restrictive disease, and in contrast with previous studies in obstructive patients with COPD [20] and asthma [22].

FrC and its corresponding fractional-order parameter β in controls were in the normal range (Fig 1C and 1D) [25, 26]. The modifications observed in these indexes must be interpreted with care. Although the small increase observed in the fractional-order compliance FrC may appear to contradict the restrictive characteristic of SCA, the reduction observed in β describes a reduction of the influence of the FrC in the elastic energy sense (reactive component), with a concomitant increase of the influence of this parameter in the dissipative energy sense (resistive component). Thus, these findings are consistent with the typical restrictive pattern associated with SCA [44] and describe pathological changes similar to those related with the reduction in Cdyn described in the first part of this study [28]. Consistent to this hypothesis, FrC was directly related with pulmonary volume parameters, such as FVC (Table 1), FRC (Table 2), and AV (Table 3).

Increased restriction in SCA patients resulted in increased values of G (Fig 1E). These findings are consistent with previous studies in patients with asbestosis [25] and may reflect the presence of increased energy dissipation in the respiratory system [17]. This effect may be explained, at least in part, by the increase in parenchymal distortion associated with interstitial pulmonary fibrosis due to the repeated episodes of ATS. Another possible explanation is related to the increase in airflow heterogeneity throughout the lung due to changes in peripheral compliance and resistance. The good correlation observed with parameters related to obstruction and restriction (Tables 1 and 2), as well as pulmonary diffusion (Table 3), provide experimental support to this hypothesis. In addition, the inverse associations between Pe (Table 4) and six minute walking test distance (6MWTD) and final SpO_2_ (Table 5) are in close agreement with these physiological interpretations.

The values of H in controls (Fig 1F) were in the same range observed in previous studies [25, 26]. This parameter was significantly reduced in SCA patients in comparison to controls. This change was not related to spirometric (Table 1) or plethysmographic indexes of restriction and airway obstruction (Table 2). These findings may reflect the inability of this parameter to describe the main effects of SCA pathophysiology.

The results observed for η in controls (Fig 1G) were consistent with that obtained in previous studies [25, 26]. The presence of restriction introduced an increase in η, which is in close agreement with previous studies [16, 25, 45] and reflects increased structural changes and heterogeneity in the lungs. This parameter is associated with the work of breathing [20], proportional to the area in the hysteresis of the pressure–volume loop and is associated with the heterogeneity of the lung tissue. Table 1 provides support for this interpretation describing the presence of inverse and good correlations with spirometric indexes of obstruction and restriction. Similar findings were observed in plethysmographic analysis (Table 2). As expected due to its physiological meaning, η presented the highest correlation among all of the studied FrOr parameters and pulmonary diffusion capacity, presenting good inverse correlations with DLCOa (%) and AV (%) (Table 3). This parameter also showed the highest correlation with functional exercise capacity. The closer associations were observed with final SpO_2_ and Borg Scale (Table 5).

These findings indicate that η clearly describes the pathophysiology of SCA, which includes reduced functional exercise capacity associated with restrictive functional defects [46] and increases in respiratory work.

In general, the correlations among the FrOr parameters and spirometry (Table 1), plethysmography (Table 2), pulmonary diffusion (Table 3), respiratory pressures (Table 4) and functional capacity (Table 5) were reasonable to good. This may be explained, at least in part, by the different conditions observed during the measurements; while spirometry and plethysmography are performed using maximal effort maneuvers, the exams used in FrOr modeling are conducted using spontaneous ventilation. Spirometry and plethysmography provide information concerning airflow and volumes, while FrOr modeling provide parameters describing with respiratory hysteresivity, damping, etc. Although these parameters are related, they are of different nature. In fact, these reasonable to good values of correlation were expected, and represents an interesting finding. It confirms that FrOr parameters can provide additional information on the mechanical characteristics of the respiratory system, which are complementary to spirometry and plethysmography, each method providing unique information.

Perhaps more important is the contribution of this analysis to our understanding of the interplay among the changes in the new FrOr parameters, the pathophysiological changes and the performance of the patients in daily life activities. We demonstrated that respiratory FrOr parameters are linked to underlying structural (Tables 1 and 2) and functional changes in the systems that produce them (Table 5). These parameters presented changes that were highly consistent with the pathophysiological changes. For example, the increase in η (Fig 1G) describes an increase in airway resistance and a decrease in pulmonary volumes (Tables 1 and 2), diffusion capacity (Table 3), respiratory muscle performance (Table 4) and functional exercise capacity (Table 5). This analysis contributes to elucidating an important debate in the literature [18, 21] and to provide support for the use of FrOr parameters in respiratory diseases.

There is general agreement in the literature about the importance of developing new, noninvasive and sensitive lung-function methods for the early and accurate detection of pulmonary abnormalities [47–50]. To contribute in this direction, in the particular case of SCA, the first part of this study initially investigated the clinical use of traditional FOT parameters [28]. This analysis considered AUCs > 0.75 to be a good cut-off value for a useful clinical discriminator and that values between 0.90 and 1.00 indicate high diagnostic accuracy [31–33]. Analysis using LOOCV showed that none of these traditional parameters reached adequate values of diagnostic accuracy (Fig 4). An important improvement was obtained using a combination of FOT and machine learning classifiers (Fig 4, AUC = 0.82 and AUC = 0.86 in normal and abnormal spirometry, respectively). Both classifiers achieved an appropriate value for clinical use (AUC>0.75) [28].

In the second part of this study, using a new and different analytical approach (FrOr modeling), we describe an additional improvement in diagnostic accuracy (Fig 4). FrOr modeling allowed us to achieve values indicating adequate (H = 0.79, Fig 2A) and high diagnostic accuracy (FrL = 0.93, β = 0.98 and η = 0.98) in SCA patients with normal spirometry. In patients with abnormal spirometry, three parameters presented high diagnostic accuracy (FrL = 0.91, β = 0.93, η = 0.93, Fig 2B), suggesting that FrOr parameters may be useful in the pulmonary function analysis of patients with SCA. These improvements may be associated with an enhanced description of the complex biomechanics of the respiratory system. Similar improvements were observed in previous studies in which FrOr modeling led to a more detailed description of the properties of the arterial wall in brain aneurysms [51] and improved the analysis of mechanics of the red blood cell membrane [52] and the simulation of blood flow in the cranial network [53].

Another interesting finding of the present work was that FOT associated with FrOr modeling resulted in parameters with higher sensitivity than spirometry in detecting an initial decline in lung function of patients with SCA (Fig 3A). FrOr parameters were also more accurate than the traditional ones in patients with abnormal spirometric exams (Fig 3B), which provides additional evidence of the usefulness of FrOr modeling in diagnostic purposes. These results are in close agreement with recent studies in which the use of fractional-order dynamics provided a significant improvement in peripheral arterial disease screening for hemodialysis patients [54], cancer detection [55], differentiation of low- and high-grade pediatric brain tumors [56] and the differentiation between malignant and benign breast lesions detected on X-ray screening mammography [57].

It has been previously indicated that recurrent fractal geometry may lead to the appearance of fractional-order terms, with the intrinsic property of phase constancy [19]. Thus, fractional-order dynamic behavior may be linked to fractal structure, implying that properties of both structure and function are fundamentally linked [58]. The bronchial tree is a highly complex fractal structure, in which the presence of self-similarity in its spatial structure is closely related to the healthy lung, whereas a diseased lung contains considerable inhomogeneities and thus asymmetry. A typical patient with SCA shows a loss of complexity in its spatial structure associated with architectural remodeling throughout its length; many segments show marked tapering, irregular constrictions, longitudinal ridges, and surface protrusions. Previous studies from our group have demonstrated a reduction in respiratory impedance complexity [59] and a significant increase in η and G with airway obstruction in asthma [24], indicating that η and G are inversely related with respiratory complexity. Thus, we can hypothesize that the increase observed in these parameters (Fig 1) may be explained, at least in part, by the progressive reduction in the complexity of the spatial structure of the airway tree of patients with SCA. This finding is consistent with the involved physiology as η is associated with the lung heterogeneity. Considering the good diagnostic performance observed in these parameters (Figs 2 and 3), this fact provides additional evidence of the clinical utility of the analysis of lung complexity reduction in the diagnosis of respiratory diseases.

The classifiers algorithms used in the development of the clinical decision support system (Table 7) have been successfully applied in respiratory research [60–65]. In the present study, experiments show that the FrOr parameters alone present a high capability in discriminating the control subjects from the patients with sickle cell anemia with normal and abnormal spirometric exams (Fig 2). Particularly, the parameters FrL, β and η present AUCs higher than 0.9, which provides a high diagnostic accuracy level. The experiments with the machine learning algorithms have shown a minor improvement (AUC = 0.97) in detecting respiratory alteration in patients with abnormal spirometric exams (Table 7). It is important to note that the dataset is small and the algorithms would require more data or a reduction in complexity, which could be accomplished with a search for the best parameters (Table 8). When the ML algorithms use a small number of the best parameters, their results were improved (AUC = 0.99, Table 8, Fig 4). Additionally, the chosen parameters for all classifiers usually include FrL, β or η, which have presented the best performance alone. As expected, the use of cross products did not improve the results. When we performed a search for the best parameters in the cross products, the results were not better than those observed using a search for the best parameters due to the small dataset and the increased complexity when one uses the cross products.

Clarifying the limitations of the present study allows the reader to better understand under which conditions these results should be interpreted. First, the present work is limited to patients with hemoglobin SS. This focus allowed us to exclude possible confounding factors regarding severity and clinical profile. Another important point is that it is often the most severe and common type of SCD. Many other types of SCD exhibit disparate features, including different structural changes within the respiratory system. Therefore, further studies are needed to assess these specific disorders.

One could argue that there is a higher number of females in the control group in relation to the groups of patients (Table 1, first part of the study [28]). However, the analyzed groups can be considered homogeneous because height is the determinant parameter in FOT analysis, and this parameter is homogeneous among the studied groups.

The subjects were from a Brazilian population at a single practice site, which affects the study’s generalizability. Therefore, multicenter studies are necessary in the future to expand the generalizability of these findings. The study used broad inclusion criteria and was performed in a typical setting under usual clinical procedures, which enhanced its generalizability. Another important point in this sense is that interested researchers may evaluate if they are likely to obtain similar outcomes in their own patient population analyzing the adopted inclusion and exclusion criteria and the demographic characteristics of the used population.

The present study investigated a relatively small sample size. Although this limitation was minimized using the LOOCV method, it is still a limitation, and additional studies including a larger number of subjects are necessary.

## Conclusion

Using a combination of FOT and fractional-order modeling, this work initially improved our knowledge regarding the respiratory changes in adults with SCA. It was shown that this disease introduces an increase in fractional inertance, damping and hysteresivity. Then, the physiological and functional meaning of the fractional-order parameters were investigated, and showed that FrL, η and β are associated with functional exercise capacity, pulmonary diffusion, respiratory muscle performance, pulmonary volumes and airway obstruction. Finally, we demonstrated that fractional-order modeling led to a high diagnostic accuracy in the detection of early respiratory abnormalities in patients with SCA, outperforming standard FOT analysis and spirometric measurements. A combination of ML methods with fractional-order modeling further improved diagnostic accuracy, composing a potentially useful clinical decision support system to help identify respiratory changes in patients with SCA.
